# Spatial analysis and factors associated with leptospirosis in Santa Catarina, Brazil, 2001-2015

**DOI:** 10.1590/0037-8682-0466-2020

**Published:** 2020-12-11

**Authors:** Ana Elisa Pereira Silva, Gleice Margarete de Souza Conceição, Francisco Chiaravalloti

**Affiliations:** 1 Universidade de São Paulo, Faculdade de Saúde Pública, Departamento de Epidemiologia, São Paulo, SP, Brasil.

**Keywords:** Leptospira, Incidence, Natural disasters, Linear regression, Spatial distribution

## Abstract

**INTRODUCTION:**

Leptospirosis is an endemic disease in Brazil that can become an epidemic during the rainy season resulting from floods in areas susceptible to natural disasters. These areas are widespread in Santa Catarina, particularly in the coastal region. Therefore, the objective of this study was to identify environmental, climatic, and demographic factors associated with the incidence of leptospirosis in the municipalities of Santa Catarina from 2001 to 2015, taking into account possible spatial dependence.

**METHODS:**

This was an ecological study aggregated by municipality. To evaluate the association between the incidence of leptospirosis and the factors under study (temperature, altitude, occurrence of natural disasters, etc.) while taking into account spatial dependence, linear regression models and models with global spatial error were used*.*

**RESULTS:**

Lower altitudes, higher temperatures, and areas of natural disaster risk in the municipality contributed the most to explaining the variability in the incidence rate. After taking spatial dependence into account, only the minimum altitude variable remained significant. The regions of lower altitude, where the highest rates of leptospirosis were recorded, corresponded to the eastern portion of the state near the coastal region, where floods, urban floods, and overflows are common occurrences. No associations were found concerning demographic factors.

**CONCLUSIONS:**

The incidence of leptospirosis in Santa Catarina was associated with environmental factors, particularly low altitude, even when considering the spatial dependence structure present in the data. The spatial error model allowed for adequate modeling of spatial autocorrelation.

## INTRODUCTION

Leptospirosis is a bacterial infection that mainly affects certain species of rodents and wild and domesticated animals. Human cases result from contact with the urine of infected rodents directly or indirectly, such as exposure to trash, debris, sewage, water, or contaminated mud. In particular, in the presence of overflows, overland flow floods, or urban floods, these bacteria spread rapidly through water and, once in contact with the skin or mucous membranes, penetrate the organism, causing infection^1^.

This disease has acquired social and economic importance due to its impact in several areas, from the financial costs of hospitalizations and treatments and years of lost life potential to its impact on the international trade of animal products^2^. In 2007, the cost of patients hospitalized with leptospirosis in Brazil’s public health system was R$ 831,500.00 (US$ 439,900.00). The life lost as a result of the disease was 15 days of life/1,000 inhabitants, and, in the case of death, the loss was 30 years/death on average^3^. The lethality of leptospirosis cases in Brazil can reach 40% in the most severe cases^4^.

Leptospirosis is considered an endemic disease that affects the entire country of Brazil; however, it can become an epidemic during the rainy season, especially in urban areas, due to flooding in areas susceptible to natural disasters^5^ ^,^ ^6^.

From 2001 to 2016, only three states did not report any cases of the disease: Roraima, Tocantins, and Piauí. In the accumulated period, Santa Catarina (SC), in the southern region, was the state with the third highest incidence of leptospirosis in Brazil, after only Acre and Amapá, both of which are located in the northern region^7^.

The state of SC, particularly its coastal region, presents geomorphological characteristics that favor the occurrence of natural disasters, such as overflows, urban floods, and mass movements. Climatic, geological, and land use conditions contribute to the increased risk of these events. Overflows and urban floods constitute 43% of the most recurrent disasters in the state^8^, while mass movements, including landslides and earthflow/mudflow, are also common. According to the Brazilian Atlas of Natural Disasters, SC had the second highest incidence of overflows in Brazil between 1991 and 2012^9^. During this period, urban floods were more frequent in the eastern part of the state, where the Itajaí-Açu River basin is located. Indeed, in 2008, this region was the site of one of the largest disasters in SC to date^10^ ^,^ ^11^.

It is essential to study the climatic and environmental factors that are potentially associated with vector-borne diseases such as leptospirosis to understand their dynamics in a particular region and how these factors influence their occurrence. One statistical method widely used for this purpose is the fit of regression model, which includes generalized linear models. A common feature of geographic data that may lead to a loss of explanatory power in statistical inference is the autocorrelation or spatial dependence which affects the incidence of the disease, generating bias in the estimates and testing hypothesis. To overcome this limitation, the regression models used for the geographic data analysis were performed to incorporate the spatial data structure^12^.

The objective of this study was to identify environmental, climatic, and demographic factors associated with the incidence of leptospirosis in the SC municipalities from 2001 to 2015, taking into account possible spatial dependence.

## METHODS

### Study area

**FIGURE 1:**
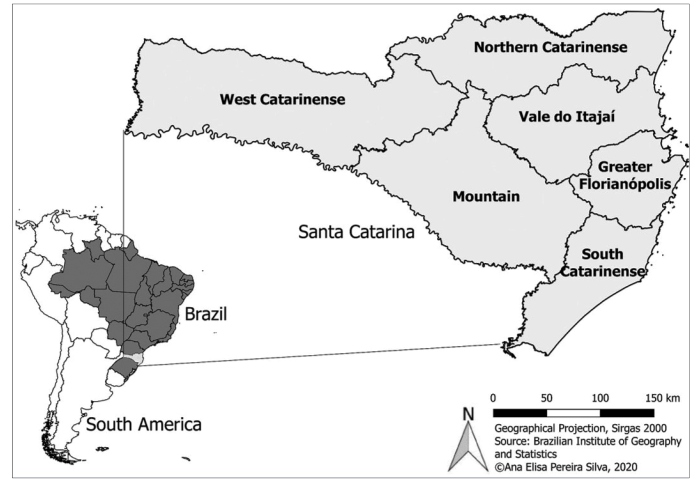
Location of the study area in the mesoregions of Santa Catarina, Brazil, South America.

Located in the southern region of Brazil, the state of SC is currently divided into 295 municipalities constituting a total area of 95,730,921 km²^13^. In this study, 293 municipalities were analyzed, since two were only emancipated in 2013. The region is predominantly subtropical and is subject to variations according to regional relief. Frost and snow are relatively common in the western portion of the state, while the climate is warmer on the coast and can reach high temperatures during the summer. Figure 1 shows the geographical location of the state of SC and its division into mesoregions in Brazil and South America.

### Disease and resident population data

Annual information on the number of confirmed cases of leptospirosis and the size of the resident population according to gender and age range for the period from 2001-2015 was obtained from the Department of Informatics of the Brazilian Health System (DATASUS) website for each municipality in SC. Population data were obtained from the 2010 Demographic Census, and estimates for the other years were made by the Interagency Health Information Network (RIPSA). 

### Incidence rate of leptospirosis

The incidence rate of leptospirosis during the study period in each municipality was obtained using the direct standardization method, standardized by gender and age group. The SC population in 2010 was used as the standard. The standardization method aims to avoid the influence of variations in age and gender composition in the population and allows for direct comparisons of incidence between municipalities.

### Climatic and environmental data

The air temperature data were acquired from the Climate Prediction Center (CPC) (www.cpc.ncep.noaa.gov), which belongs to the National Oceanic and Atmospheric Administration (NOAA). Based on daily observations of the land surface and interpolation at a 0.5-degree grid point for the entire globe^14^, maximum and minimum air temperature data were obtained, and from these data, the average temperature. Minimum, mean, and maximum annual values were extracted closer to the geographical location of the municipalities of SC, and the average value of each variable was calculated for the study period.

The altitude data were obtained from the Brazilian Geomorphometric Data Bank (Topodata) (www.dsr.inpe.br/topodata/), which offers products for the Brazilian territory, prepared from raster images of the Shuttle Radar Topographic Mission (SRTM) made available by the United States Geological Survey (USGS). Using the Shapefile vector files, the minimum, mean, and maximum altitudes were calculated for each SC municipality.

Information on natural disasters occurring in Brazil is recorded by the National Secretariat for Civil Protection and Defense (SEDEC) according to the Brazilian Classification and Codification of Disasters (COBRADE) and provided by the Integrated Information System on Disasters (S2ID). The incidence of natural disasters in each SC municipality during the study period was obtained. Only disasters considered capable of carrying the *Leptospira* bacteria present in the soil were selected: landslides, earthflow/mudflows, overflows, urban floods, overland flow floods, and heavy rains. A disaster could involve the presence of one or more of these events simultaneously.

The municipalities of SC with a historical risk of natural disasters were mapped by the Brazilian Geological Survey, also known as the Mineral Resource Research Company (CPRM), to indicate possible areas of overflow, overland flow flood, urban flood, and mass movement occurrence. Some municipalities use their own risk mapping. These data were provided by the National Center for Natural Disaster Monitoring and Alerts (Cemaden) and used to determine the existence and size (%) of risk areas mapped to hydrological or geological disasters in the municipalities.

Population and land area data for 2010 were used to calculate the demographic density of the municipalities (inhabitants/km²) using QGIS software. The proportion of the resident population living in the urban area in relation to the total population of the municipality was obtained from the 2010 Demographic Census results available from the Brazilian Institute of Geography and Statistics (IBGE).

### Statistical analysis

Initially, to evaluate the association between the incidence of leptospirosis in SC and climatic, environmental, and demographic factors, a multiple linear regression model was fitted. The dependent variable was the natural logarithm of the rate of leptospirosis in each municipality. As some municipalities did not have cases of leptospirosis, before the logarithmic transformation, a value of "one" was added to the rates of all municipalities. The independent variables were the minimum, mean, and maximum temperature, minimum altitude, mean and maximum altitude, number of natural disasters, existence of risk areas for natural disasters (yes/no), proportion of the size of the risk area in relation to the size of the municipality (%), population density (inhabitants/km²), and proportion of residents living in urban areas (%) in each municipality. Given the large number of explanatory variables, the *stepwise forward* method was used. First, simple regression models were fit to each explanatory variable. Next, the variables were sequentially inserted into the model according to the coefficient of determination (R^2^) of the simple model (from highest to lowest). The criterion for deciding whether a variable should remain in the model was a p-value < 0.10 for the t-test for its coefficient and a p-value < 0.05 for the likelihood ratio test (which compares the model with the variable versus the model without the variable).

After obtaining the final model, a residue analysis was performed based on the graphs between the residuals and the fitted values and standardized residuals (Normal Q-Q). Due to the nature of the data geographically distributed in space, it was possible that the model would be unable to explain the spatial dependence of the incidence of the disease among municipalities, resulting in dependence on the residuals. To evaluate this assumption, Moran’s index was used, which indicates how the values are correlated in space^12^ by testing the null hypothesis of spatial independence against the alternative hypothesis of spatial dependence. This index was obtained from the construction of the neighborhood matrix by queen contiguity among the municipalities, which considers the municipalities that have borders with each other as neighbors, to which the same weight is assigned. Each municipality was related, on average, to five neighbors.

Considering the presence of spatial autocorrelation, models that consider the correlation structure and incorporate it as a parameter in the generated linear regression model were used. These models have global spatial effects: the *spatial lag* model, which assigns spatial correlation to the dependent variable and the *spatial error* model, which considers the spatial correlation as noise (error) to be removed^15^. The steps of the Lagrange multiplier test decision process were followed to select the best model to use^16^. After fitting the selected model, the residues were retested using Moran’s index to determine if the spatial dependency was eliminated. Normality was assessed using the residual graph (Normal Q-Q).

The analysis was performed with the aid of R and QGIS software, version 2.18.

## RESULTS

**FIGURE 2:**
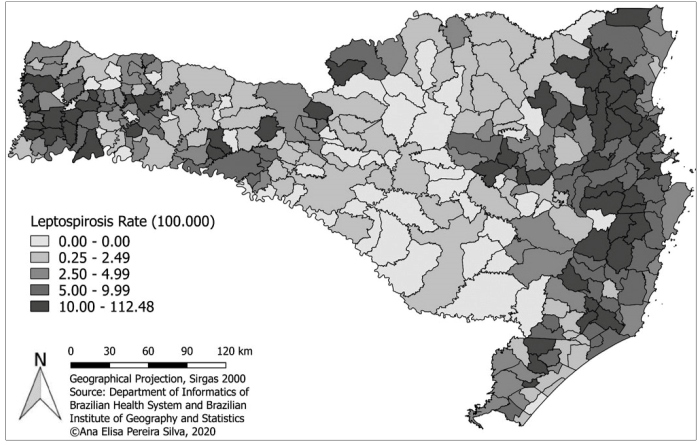
Incidence rate of leptospirosis in the municipalities of SC, classified by quantile, from 2001 to 2015.

Figure 2 shows a map with incidence rates of leptospirosis from 2001-2015 in all municipalities of SC. The highest incidences were found in the eastern and western portions of SC, indicating clusters of municipalities with similar incidence rates and a possible spatial correlation between them. The number and percentage of municipalities with zero incidence was 51 (17%) in relation to the total number of municipalities.

Table 1 shows the estimates of the simple regression models. Apart from resident household population in urban areas, there was a significant association between the incidence rate of leptospirosis and the other variables, and the minimum altitude variable had the highest R².

**TABLE 1:** Estimates of the simple linear regression models.

Variables description	Beta coefficient	Standard error	p-value	R²
Minimum temperature (°C)	0.25	0.03	<0.001	0.16
Mean temperature (°C)	0.26	0.04	<0.001	0.13
Maximum temperature (°C)	0.18	0.04	<0.001	0.07
Minimum altitude (m)	-0.002	0.0002	<0.001	0.24
Mean altitude (m)	-0.001	0.0002	<0.001	0.17
Maximum altitude (m)	-0.0005	0.0002	<0.01	0.03
Number of natural disasters	0.03	0.02	<0.05	0.01
Existence of risk area (yes/no)	0.50	0.14	<0.001	0.04
Size of risk area (%)	0.08	0.03	<0.05	0.02
Demographic density (inhab/km²)	0.0006	0.0004	0.05	0.01
Residents in the urban area (%)	0.001	0.003	0.62	0.0009

°C: degrees Celsius; m: meter; %: percent.

Table 2 shows the estimates of the adjusted linear and spatial regression model parameters. According to the final multiple linear regression model, the significant independent variables explaining the variability in the incidence rate were the minimum altitude, the maximum temperature, and the presence of an area of risk in the municipality. The residue analysis indicated the existence of some outliers, but the assumptions of normality and homoscedasticity were satisfied.

**TABLE 2:** Estimates of the multiple and spatial linear regression models

	Linear regression model		***Spatial error* model**	
	Coefficient	p-value	Coefficient	p-value
Intercept	-0.13	0.89	1.00	0.46
Minimum altitude (m)	-0.002	<0.0001	-0.002	<0.0001
Maximum temperature (°C)	0.09	0.02	0.04	0.45
Existence of risk area	0.22	0.09	0.11	0.39
R² adjusted	0.25	-	-	-
Lambda	-	-	0.52	-
AIC	779.64	-	740.76	-

Adjusted for maximum temperature and existence of risk area. **°C:** degrees Celsius; **m:** meter; **AIC:** Akaike information criterion; **Lambda:** autoregressive coefficient.

Moran’s index was 0.24 (p < 0.0001), indicating the existence of spatial dependence on the residuals and the need to use a regression model that would incorporate these spatial effects. The Lagrange multiplier test suggested two possible models: *spatial lag* (SAR) and *spatial error* (CAR). In the first stage of the decision-making process, both models were statistically significant (p < 0.0001). In the second stage, in which the diagnosis was more robust, neither model was significant. We chose the *spatial error* model, which has a lower descriptive level (p = 0.20), making it the most appropriate.

The Akaike information criterion (AIC) for this model was lower than that for the linear model, indicating a better fit, with an autoregressive coefficient (lambda) of 0.52, which represented the nonrandom part of the error. Moran’s test, which was applied to residuals of the regression model with spatial effects, indicated the elimination of the existing spatial dependence (I = 0.02 and p = 0.26). Again, normality and homoscedasticity were observed in the analysis of the residuals of this model.

**FIGURE 3:**
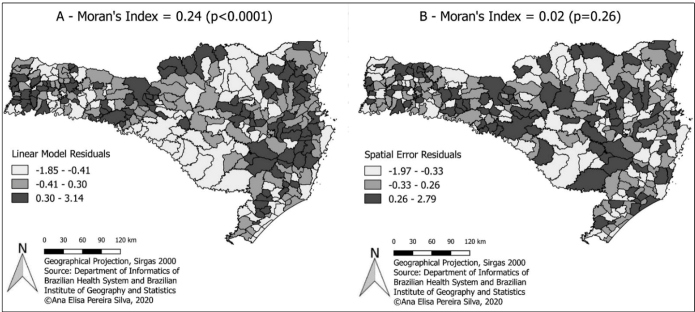
Residual maps of the linear regression **(A)** and *spatial error* **(B)** models**.**

The spatial distributions of the residuals of the linear regression and *spatial error* models are shown in Figure 3. In the linear model, Moran’s index indicated a greater number of positive residues in the eastern and western regions of SC, while the negative residues were located preferentially in the central portion of the state. In the spatial model, Moran’s index suggested a lower variability in addition to geographic randomness in the waste distribution, indicating that this model was the most appropriate. According to the model estimates, for each increase of 100 m in the minimum altitude, the risk of disease decreased by approximately 18%.

## DISCUSSION

This study investigated the association between the incidence of leptospirosis and environmental, climatic, and demographic factors in municipalities of SC. The highest incidence occurred in municipalities at the lowest altitude and with the highest temperatures, which presented as areas of risk for hydrological and mass movement events. However, when these factors were considered together to explain the variability of incidence rates and after considering the spatial data structure, only altitude remained significantly associated with incidence. This does not suggest that the disease is not influenced by the other factors mentioned, but rather that, once altitude is known, the other factors do not add additional information to explain the incidence variability. It is reasonable to consider the fact that these factors do not occur in isolation but are associated with each other. In fact, high temperatures are commonly recorded at lower elevations, meaning that temperature and altitude contain the same environmental information to a large degree^17^. Likewise, hydrological disaster risk areas, such as overflow and overland flow flooding, are located near rivers at lower-altitude sites. These areas are more conducive to human occupation because they are flatter, but this feature also favors the accumulation of water during hydrological disasters^18^.

In this study, an inverse association between the incidence of leptospirosis and a decrease in altitude was identified, that is, the disease occurred more frequently at lower-altitude sites. This result is consistent with several other studies in Brazil and worldwide^19^ ^-^ ^22^.

Barcellos et al.^23^ (2003) analyzed the distribution of leptospirosis cases in the municipalities of Rio Grande do Sul, Brazil, in 2001. The state was divided into three altitude ranges (0-300, 300-800, and > 800 m), and the authors found that 85.6% of the cases were in lower altitude areas, while at higher altitudes, the rates were significantly lower than the mean.

In another study in Rio Grande do Sul between 2008 and 2012, Schneider et al.^24^ (2015) concluded that a large number of cases of leptospirosis were associated with the interaction between altitude and slope. The highest rates of leptospirosis were recorded in the municipalities of the central and northern areas of SC, especially those at lower altitudes. Urban cases were more concentrated in the metropolitan areas of municipalities at low altitudes, including the state capital, which experiences frequent flooding.

Péres et al.^25^ (2019) studied the rate of hospitalization for leptospirosis in SC and its association with extreme hydrological events such as overflowing from 2005-2014. Higher hospitalization rates and numbers of extreme hydrological events (with peaks in the summer months) were found in coastal regions, where altitudes are lower, than in the interior of the state. 

In the present study, the regions where the highest rates of leptospirosis were recorded corresponded to the entire eastern portion of SC near the coastal region, which encompasses the eastern mesoregions of the Northern Catarinense and Vale do Itajaí, the coastal region of Greater Florianópolis (including the capital), and the southern mesoregion of SC. In these regions, floods are frequent, especially urban floods and overflows^8^.

In some municipalities, the incidence rate of leptospirosis for the period exceeded 100 per 100,000 inhabitants, while Brazil recorded an average incidence of 1.9 and the state of SC, 0.5 per 100,000 inhabitants.

Several studies, generally of time series, have found an association between the volume of rainfall (precipitation) and the incidence of leptospirosis. However, in a study conducted in Pelotas, a municipality located in southern Brazil, Jorge et al.^26^ (2017) found no significant association between human leptospirosis and rainfall accumulation over a period of three months. Approximately one month after the occurrence of heavy rains, which cause natural disasters such as overflow, urban floods, and landslides, there is usually a significant increase in the number of cases of leptospirosis^11^ ^,^ ^27^. Therefore, this study, in which a spatial analysis was performed, did not evaluate the contribution of rain directly, but rather the existence of areas at risk for disasters, such as overflow, urban flooding, and landslides, caused by large rainfall volumes in short periods.

In this study, clusters of municipalities had similar rates, suggesting that the rates of leptospirosis in a given municipality are correlated with the rates of their close neighbors. Although the territorial limit indicates separation between municipalities, these municipalities are often linked by waterways (rivers, streams, etc.), which are important disseminators of *Leptospira interrogans* bacteria. Diesel and Ometto^28^ (2016) observed that, in 2008, the municipalities with the most reported cases of leptospirosis in SC were located in areas with frequent overflows caused by river elevation and consequent overfilling, reaching more than one municipality simultaneously. Just as the distribution of disease shows a dependence between close neighbors, factors associated with the occurrence of disease should also exhibit such geographical behavior, such as environmental, climatic, and demographic variations in space.

For several years, spatial models have been applied in ecological studies because the phenomenon of spatial autocorrelation, which is frequently observed in this type of study, can be modeled, allowing for the acquisition of more accurate estimates and a better fit (lower AIC) than classical regression models^29^.

Rood et al.^30^ (2017) investigated the geographic distribution of the incidence of leptospirosis in the Netherlands and its association with environmental factors favorable to the onset of the disease, per municipality, from 1995 to 2012. After observing the existence of spatial clusters of the disease that indicated spatial dependence, they used an autoregressive spatial model, or *spatial lag*, which allowed for the identification of associations between the incidence of the disease and the cover of arable land, built area, pasture, and clay soil.

The limitation of the current study was the use of secondary data, for which it was not possible to verify the quality. However, since the data used for both leptospirosis and other variables belong to public agencies, they were officially considered adequate for the proposed study. Regardless, the main strength of the study was the consideration of the spatial dependence of the studied phenomenon in the modeling.

A significant spatial autocorrelation was detected for the incidence of leptospirosis, indicating a nonrandom pattern of disease distribution throughout the municipalities; however, the *spatial error* model allowed us to account for this in the analysis. The incidence of leptospirosis in the municipalities of SC from 2001 to 2015 was associated with environmental factors, most notably, the minimum altitude. This association remained significant even after controlling for other climatic and environmental factors (such as the maximum temperature and the presence of at-risk areas for natural disasters). No associations were found with demographic factors such as the population density and proportion of the resident population living in urban areas. The highest incidence occurred in municipalities with the lowest altitudes, after considering spatial dependence present in the geographic data.
